# Molecular characterization of clinical non-typhoidal *Salmonella* isolates shows high antimicrobial resistance burden in Jiangsu, China

**DOI:** 10.3389/fmicb.2025.1587421

**Published:** 2025-05-30

**Authors:** Hui Cao, Yun Shen, Kai Ma, Dongyu Zheng, Yan Xu, Xin Qiao

**Affiliations:** ^1^Department of Nutrition and Food Safety, Jiangsu Provincial Center for Disease Control and Prevention, Nanjing, China; ^2^Jiangsu Provincial Medical Key Laboratory of Pathogenic Microbiology in Emerging Major Infectious Diseases, Nanjing, China

**Keywords:** non-typhoidal *Salmonella*, antimicrobial resistance, whole genome sequencing, serotyping, plasmid-mediated resistance

## Abstract

Non-typhoidal *Salmonella* (NTS) poses a significant global health burden due to its association with gastroenteritis and rising antimicrobial resistance (AMR). This study conducted a genomic analysis of 62 *Salmonella* isolates from outpatient cases in Jiangsu, China, to monitor the epidemiological characteristics of NTS, including genetic diversity, AMR profiles, and resistance transmission mechanisms 18 serovars and 21 sequence types (STs) were identified by whole genome sequencing, with *S. enteritidis* (27.42%) and *S. typhimurium* (19.35%) predominating. 61 resistance genes from ten different antimicrobial categories were found by genotypic AMR screening. 90.32% of isolates had *β*-lactam resistance genes, indicating a high frequency of extended-spectrum β-lactamases (ESBL). Serovar-dependent resistance patterns were highlighted by the most varied AMR profile (40/61 genes) found in *S. typhimurium*. The co-occurrence of genes for aminoglycoside resistance, *sul2*, and *blaTEM* indicated clustering driven by mobile genetic elements. A plasmid in a *S. Stanley* isolate harbored 12 AMR genes, which showed structural changes suggestive of horizontal gene transfer and active recombination. These findings underscore the role of plasmids in disseminating MDR and the urgent need for enhanced antimicrobial stewardship, food safety protocols, and One Health interventions to mitigate the spread of resistant *Salmonella* clones.

## Graphical abstract

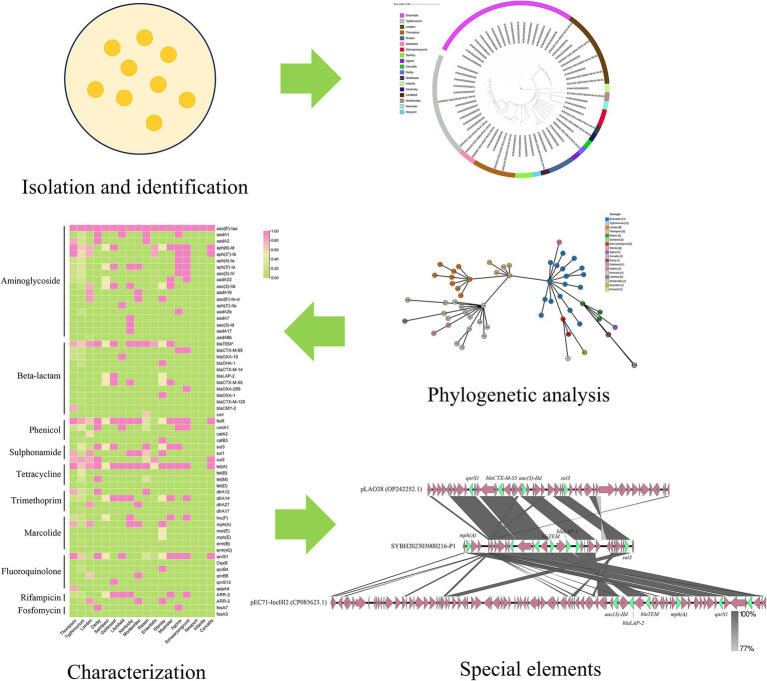

## 1 Introduction

*Salmonella* is a Gram-negative facultatively anaerobic bacterium, belonging to the Enterobacteriaceae family. *Salmonella* Genus is highly diversity, more than 2,600 *Salmonella* serovars have been described to date (Guibourdenche et al., 2010). Acute gastroenteritis is frequently caused by non-typhoidal *Salmonella* (NTS) all over the world (Alikhan et al., 2018). Even though the majority of human cases of salmonellosis are mild and self-limiting, dehydration brought on by severe vomiting and diarrhea can occasionally be fatal. Every year, NTS causes over 180 million instances of gastroenteritis and over 300,000 fatalities worldwide (Kirk et al., 2015; Besser, 2018). Salmonellosis incidence was estimated to be 626.5 cases per 100,000 people in China, where NTS is the second most frequent bacterium causing foodborne outbreaks (Chen J. et al., 2024). *Salmonella* might contaminate animal carcasses during transport, slaughtering, and then transmitted to humans via the farm-to-fork route, causing severe infections and threatening public health systems (Pan et al., 2018).

Treatment of bacterial infections is severely hampered by the misuse of antimicrobial drugs. *Salmonella* poses significant risks to public health and security because it is thought to be a significant reservoir of genes that cause antibiotic resistance (Jajere, 2019). Effective preventative and dietary control measures have previously reduced the general incidence of NTS; nevertheless, the emergence of multidrug-resistant clones in recent years has resulted in a large rise in the burden of human NTS infection (Xiang et al., 2020; Wang et al., 2020). Resistance to *β*-lactam antibiotics has increased over the past 20 years, and in some countries, contaminated food products have been shown to contribute to the spread of *Salmonella* isolates that produce extended-spectrum *β*-lactamase (ESBL) and the resulting health risks (Balta et al., 2024). The occurrence of multi-drug resistant bacteria lead to more complicate situation in *Salmonella* infection treatment and is regarded as a serious threat to public health because it limits the options for therapeutic treatment of patients (Collignon, 2013). *Salmonella* isolates recovered from animal farms, food chain processes, foods, and humans have been found to be resistant to various antibiotics (Elbediwi et al., 2019; Klemm Elizabeth et al., 2018).

Strategies for reducing the spread of disease will make more sense if the risk factors linked to bacterial infections are understood. In order to enhance our understanding of NTS infections, we used whole genome sequencing to examine the genetic diversity, MLST, antimicrobial resistance, virulence genes, and serotype of *Salmonella* isolates obtained from outpatients in Jiangsu province, China.

## 2 Materials and methods

### 2.1 Collection and identification of bacterial isolates

A total of 62 *Salmonella* isolates were isolated from the feces of patients with clinical diarrhea. These patients were from sentinel hospitals for foodborne diseases in Jiangsu Province. The suspected cause of the disease was the consumption of contaminated food. Diarrhea is defined as having three or more bowel movements within 24 h, accompanied by abnormal stool characteristics such as watery stool, loose stool, or mucus stool. MacConkey and *Salmonella* and *Shigella* (SS) plates were used to identify and purify those isolates, suspected *Salmonella* colonies on SS agar were 1–2 mm in size, circular, transparent, smooth and with a black centre, the colonies will be cultured in LB and the species of all isolates were confirmed as *Salmonella* by using mass spectrometry at first (Kim et al., 2022).

### 2.2 Genomic DNA extraction and sequencing

Genomic DNA of all *Salmonella* isolates was extracted by using FastPure Bacterial DNA Isolation Mini Kit (Vazyme, China) following the manufacturer’s instructions. Whole genome sequencing was performed on the Illumina NovaSeq PE150 platform, and the quality of the raw data was controlled by using fastQC,^1^ and filtered by using Trim-galore,^2^ followed by *de novo* assembly using SOAP denovo (version 2.04) (Li et al., 2010; Li et al., 2008). The quality of these genome files was assessed by checkM2 (Chklovski et al., 2023).

### 2.3 Serotyping and MLST

The serovar, serogroup and O antigen of the *Salmonella* isolates was predicted by using SISTR (Salmonella *In Silico* Typing Resource) (Yoshida et al., 2016). Multilocus serotyping was performed by using mlst.^3^

### 2.4 Genome wide phylogenic analysis

Whole genome phylogenetic tree was constructed by using Mashtree (Katz et al., 2019), and visualized by iTOL (Letunic and Bork, 2019). cgMLST was performed to further analyze the phylogenetic difference between these *Salmonella* isolates, all genomes were annotated by Prokka (Seemann, 2014), and the core genome was collected from the result of Roary (Page et al., 2015), then Bac_cgMLST^4^ was used to perform the cgMLST. A minimum spanning tree was generated to analyze the relationship between genomic characteristics and the typing results by using GrapeTree (Zhou et al., 2018).

### 2.5 Antimicrobial resistance genes analysis

Antimicrobial susceptibility of these *Salmonella* isolates was tested following Clinical & Laboratory Standards Institute (CLSI) 2021 using VITEK® 2 COMPACT (bioMérieux). The following antibiotics were used in this study: ampicillin (AMP), Ampicillin-Sulbactam (AMS), imipenem (IPM), tetracycline (TET), gentamicin (GEN), chloramphenicol (CHL), Colistin (CT), azithromycin (AZM), cefotaxime (CTX), ceftazidime (CAZ), cefazolin (CFZ), cefoxitin (CFX), ciprofloxacin (CIP), and nalidixic acid (NAL).

The present of antimicrobial resistance genes (AMRGs) in every genome of *Salmonella* isolates was predicted by using ResFinder (Bortolaia et al., 2020), the threshold was identity > 90%, coverage > 60%. The result was organized and a headmap was built by using TBtools (Chen et al., 2020). Further, the frequency of the combinations of AMRGs were calculated.

### 2.6 Scanning the plasmid replicons

The assembled contigs were then used to predict plasmid replicons and by using PlasmidFinder 2.1,^5^ the threshold was identity > 95%, coverage > 60%.

### 2.7 Comparison of the antimicrobial resistance gene carrying elements

To further discuss the hazard of highly burden of AMRGs, the elements carrying the greatest number of AMRGs were analyzed. The genome was scaffolded by RagTag (Alonge et al., 2022), the comparison between closest elements were performed using Easyfig (Sullivan et al., 2011).

### 2.8 Statistical analysis

Basic Python calculation method was used in this study to count the number of groups. No comparison of groups was performed in this study. If not stated, all tools we used in this study were used according to default Settings.

## 3 Results

### 3.1 Population structure of *Salmonella*

62 *Salmonella* isolates have been isolated and identified from outpatients from Jiangsu province in this study (Table 1). A diverse serogroup distribution was discovered, including B, C1, C2, C3, D1, E1, F. Further, a total number of 18 serovars were identified, among them, *S. enteritidis* is predominant (17/62; 27.42%), followed by *S. typhimurium* (12/62; 19.35%), *S. London* (9/62; 14.52%) and *S. Thompson* (5/62; 8.06%) (Table 2).

**Table 1:** Information of 62 *Salmonella* isolates isolated in this study.

Isolates	Serotype	Serogroup	O antigen	Sequence type	aroC	dnaN	hemD	hisD	purE	sucA	thrA
32,011,103–2022-00124-FB-01-SM-01	Thompson	C1	6,7,14	26	14	13	18	12	14	18	1
32,011,301–2022-00115-FB-01-SM-01	Typhimurium	B	1,4,[5],12	19	10	7	12	9	5	9	2
32,011,501–2022-00190-FB-01-SM-01	Thompson	C1	6,7,14	26	14	13	18	12	14	18	1
32,021,105–2022-00032-FB-01-SM-01	London	E1	3,{10}{15}	155	10	60	58	66	6	65	16
32,021,105–2022-00094-FB-01-SM-01	London	E1	3,{10}{15}	155	10	60	58	66	6	65	16
32,028,102–2022-00073-FB-01-SM-01	Derby	B	1,4,[5],12	40	19	20	3	20	5	22	22
32,031,103–2022-00056-FB-01-SM-01	Saintpaul	B	1,4,[5],12	27	5	14	18	9	6	12	17
32,040,203–2022-00284-FB-01-SM-01	Goldcoast	C2-C3	6,8	358	5	110	35	122	2	19	22
32,058,302–2022-00073-FB-01-SM-01	Litchfield	C2-C3	6,8	214	14	72	21	12	6	19	15
32,058,302–2022-00157-FB-01-SM-01	Saintpaul	B	1,4,[5],12	49	5	14	21	9	6	12	17
32,090,201–2022-00109-FB-01-SM-01	Typhimurium	B	1,4,[5],12	34	10	19	12	9	5	9	2
32,090,202–2022-00015-FB-01-SM-01	London	E1	3,{10}{15}	155	10	60	58	66	6	65	16
32,101,201–2022-00005-FB-01-SM-01	Kentucky	C2-C3	8,20	198	76	14	3	77	64	64	67
32,111,101–2022-00076-FB-01-SM-01	London	E1	3,{10}{15}	155	10	60	58	66	6	65	16
32,118,101–2022-00053-FB-01-SM-01	London	E1	3,{10}{15}	155	10	60	58	66	6	65	16
32,132,301–2022-00138-FB-01-SM-02	Montevideo	C1	6,7,14,[54]	4	43	41	16	13	34	13	4
SYBH202303000189	Typhimurium	B	1,4,[5],12	19	10	7	12	9	5	9	2
SYBH202303000190	Typhimurium	B	1,4,[5],12	19	10	7	12	9	5	9	2
SYBH202303000191	Rissen	F	6,7,14	469	92	107	79	156	64	151	87
SYBH202303000192	Enteritidis	D1	1,9,12	11	5	2	3	7	6	6	11
SYBH202303000193	Enteritidis	D1	1,9,12	11	5	2	3	7	6	6	11
SYBH202303000194	Enteritidis	D1	1,9,12	11	5	2	3	7	6	6	11
SYBH202303000195	Typhimurium	B	1,4,[5],12	34	10	19	12	9	5	9	2
SYBH202303000196	Typhimurium	B	1,4,[5],12	34	10	19	12	9	5	9	2
SYBH202303000197	Typhimurium	B	1,4,[5],12	19	10	7	12	9	5	9	2
SYBH202303000198	London	E1	3,{10}{15}	155	10	60	58	66	6	65	16
SYBH202303000199	Enteritidis	D1	1,9,12	11	5	2	3	7	6	6	11
SYBH202303000200	Enteritidis	D1	1,9,12	11	5	2	3	7	6	6	11
SYBH202303000201	London	E1	3,{10}{15}	155	10	60	58	66	6	65	16
SYBH202303000202	Typhimurium	B	1,4,[5],12	34	10	19	12	9	5	9	2
SYBH202303000203	Stanley	B	1,4,[5],12,[27]	29	16	16	20	18	8	12	18
SYBH202303000204	Rissen	F	6,7,14	469	92	107	79	156	64	151	87
SYBH202303000205	Thompson	C1	6,7,14	26	14	13	18	12	14	18	1
SYBH202303000206	Enteritidis	D1	1,9,12	11	5	2	3	7	6	6	11
SYBH202303000207	Typhimurium	B	1,4,[5],12	34	10	19	12	9	5	9	2
SYBH202303000208	Enteritidis	D1	1,9,12	11	5	2	3	7	6	6	11
SYBH202303000209	Muenster	E1	3,{10}{15}{15,34}	321	119	10	17	42	12	13	4
SYBH202303000210	Agona	B	1,4,[5],12	13	3	3	7	4	3	3	7
SYBH202303000211	Typhimurium	B	1,4,[5],12	34	10	19	12	9	5	9	2
SYBH202303000212	Schwarzengrund	B	1,4,12,27	96	43	47	49	49	41	15	3
SYBH202303000213	Schwarzengrund	B	1,4,12,27	96	43	47	49	49	41	15	3
SYBH202303000214	Enteritidis	D1	1,9,12	11	5	2	3	7	6	6	11
SYBH202303000215	Enteritidis	D1	1,9,12	11	5	2	3	7	6	6	11
SYBH202303000216	Stanley	B	1,4,[5],12,[27]	29	16	16	20	18	8	12	18
SYBH202303000217	Enteritidis	D1	1,9,12	11	5	2	3	7	6	6	11
SYBH202303000218	Thompson	C1	6,7,14	-	14	~13	18	12	14	18	1
SYBH202303000220	Rissen	F	6,7,14	469	92	107	79	156	64	151	87
SYBH202303000221	Enteritidis	D1	1,9,12	11	5	2	3	7	6	6	11
SYBH202303000222	Newport	C2-C3	6,8,20	166	5	14	6	12	5	14	58
SYBH202303000223	Enteritidis	D1	1,9,12	11	5	2	3	7	6	6	11
SYBH202303000224	Thompson	C1	6,7,14	26	14	13	18	12	14	18	1
SYBH202303000225	Infantis	C1	6,7,14	32	17	18	22	17	5	21	19
SYBH202303000226	Enteritidis	D1	1,9,12	11	5	2	3	7	6	6	11
SYBH202303000227	Enteritidis	D1	1,9,12	11	5	2	3	7	6	6	11
SYBH202303000229	London	E1	3,{10}{15}	155	10	60	58	66	6	65	16
SYBH202303000230	Enteritidis	D1	1,9,12	11	5	2	3	7	6	6	11
SYBH202303000231	Enteritidis	D1	1,9,12	11	5	2	3	7	6	6	11
SYBH202303000232	Typhimurium	B	1,4,[5],12	19	10	7	12	9	5	9	2
SYBH202303000233	Corvallis	C2-C3	8,20	1,541	197	187	10	234	8	65	22
SYBH202303000235	London	E1	3,{10}{15}	155	10	60	58	66	6	65	16
SYBH202303000236	Typhimurium	B	1,4,[5],12	34	10	19	12	9	5	9	2
SYBH202303000237	Enteritidis	D1	1,9,12	11	5	2	3	7	6	6	11

**Table 2:** Typing details of the *Salmonella* isolates.

Groups of sero-group of *Salmonella*	Serotype of *Salmonella*	ST of *Salmonella*	Numbers of isolates
Group B (20/62; 32.26%)	Typhimurium (12/62; 19.35%)	34 (7/62; 11.29%)	7
		19 (5/62; 8.06%)	5
	Schwarzengrund (2/62; 3.23%)	96 (2/62; 3.23%)	2
	Stanley (2/62; 3.23%)	29 (2/62; 3.23%)	2
	Saintpaul (2/62; 3.23%)	27 (1/62; 1.61%)	1
		49 (1/62; 1.61%)	1
	Agona (1/62; 1.61%)	13 (1/62; 1.61%)	1
	Derby (1/62; 1.61%)	40 (1/62; 1.61%)	1
Group C1 (7/62; 11.29%)	Thompson (5/62; 8.06%)	26 (4/62; 6.45%)	4
		nem* (1/62; 1.61%)	1
	Infantis (1/62; 1.61%)	32 (1/62; 1.61%)	1
	Montevideo (1/62; 1.61%)	4 (1/62; 1.61%)	1
Group C2-C3 (5/62; 8.06%)	Corvallis (1/62; 1.61%)	1,541 (1/62; 1.61%)	1
	Goldcoast (1/62; 1.61%)	358 (1/62; 1.61%)	1
	Kentucky (1/62; 1.61%)	198 (1/62; 1.61%)	1
	Litchfield (1/62; 1.61%)	214 (1/62; 1.61%)	1
	Newport (1/62; 1.61%)	166 (1/62; 1.61%)	1
Group D1 (17/62; 27.42%)	Enteritidis (17/62; 27.42%)	11 (17/62; 27.42%)	17
GroupE1 (10/62; 16.39%)	London (10/62; 16.39%)	155 (10/62; 16.39%)	9
	Muenster (1/62; 1.61%)	321 (1/62; 1.61%)	1
GroupF (3/62; 4.82%)	Rissen (3/62; 4.82%)	469 (3/62; 4.82%)	3

* nem = no exact match.

Multi-locus sequence typing shows that 21 sequence types (STs) were identified with the dominance of ST11 (17/62; 27.42%), ST155 (10/62; 16.39%) and ST34 (7/62; 11.29%). There are 3 serovars presented multiple STs, which is *S. typhimurium* (ST34, ST19), *S. Saintpaul* (ST27, ST49), and *S. Thompson* (ST26 and 1 no exact match), and the rest of them presented only one ST (Table 2).

### 3.2 Phylogenomic analysis

**Figure 1:**
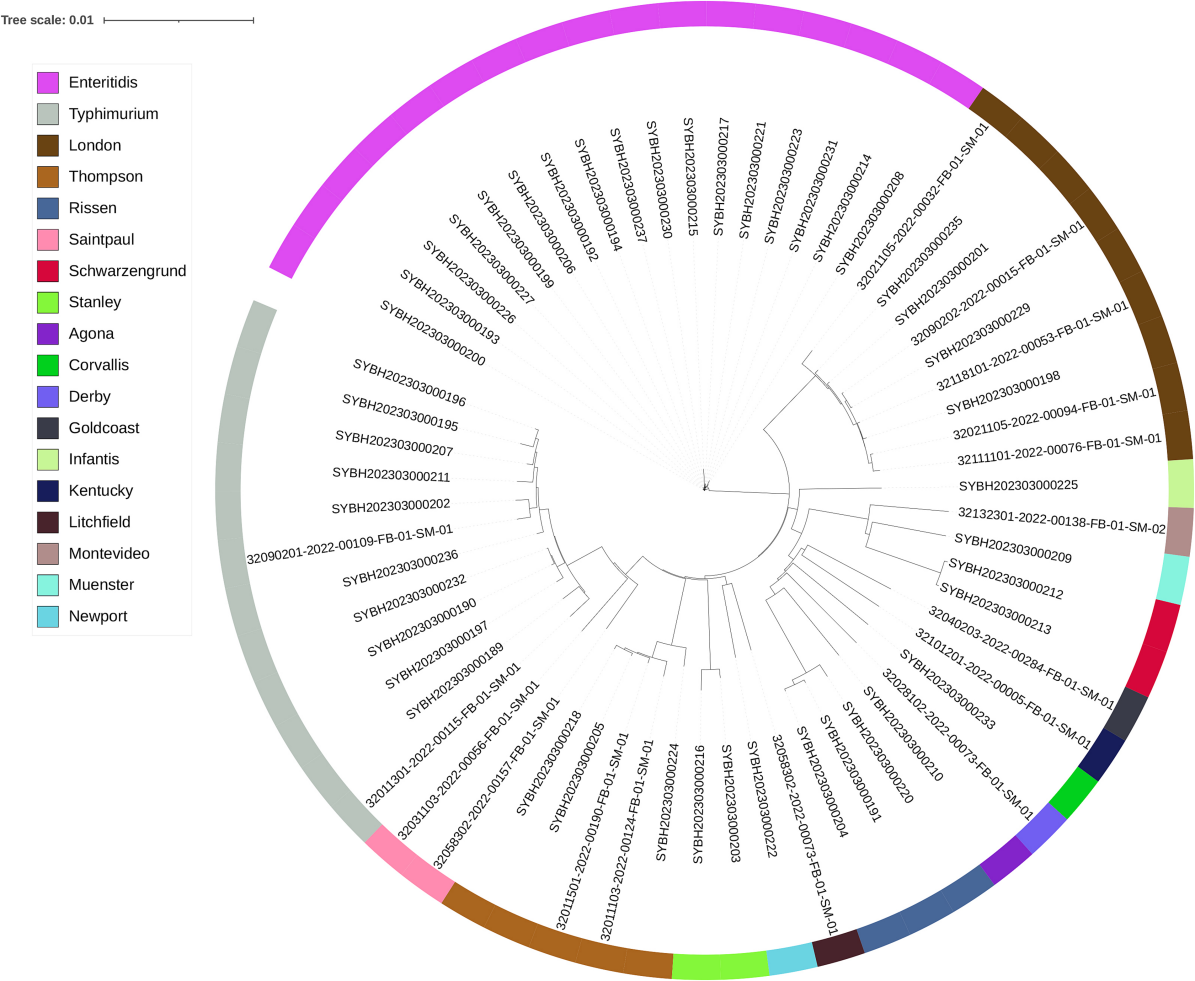
The phylogenomic tree of 62 *Salmonella* isolates constructed by Mashtree. Isolates are annotated with the serotype predicted by SISTR.

Through sequencing quality assessment, the average genomes size of those genome files is 4,905,831 bp, with a N50 between 143, 671 bp and 411,631 bp, the average N50 is 233,405 bp (Supplementary Table 1). A phylogenetic tree based on the whole genome sequence was constructed for these *Salmonella* isolates to determine their epidemiological relatedness, and for all identified serovars, the isolates belong to the same serovar is clustered in the same clade. This finding suggested the serovar of *Salmonella* was closely related to genome evolution (Figure 1).

**Figure 2:**
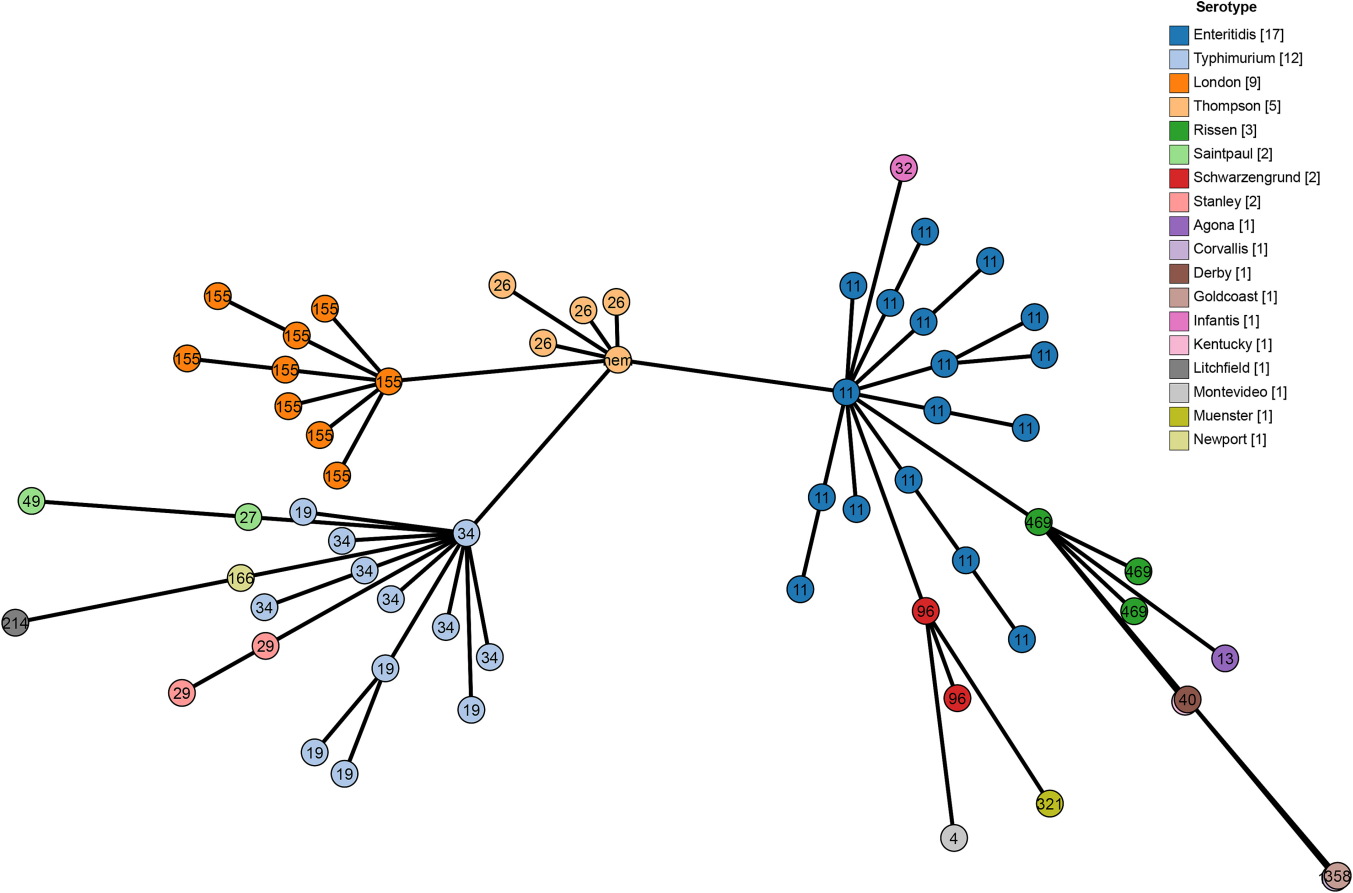
Minimum spanning tree using cgMLST profiles of 62 *Salmonella* isolates. The colors are marked by the serotype predicted by SISTR and the sequence types are marked by numbers.

A cgMLST analysis was performed to further analyze the correlationship between genome evolution and typing data, *S. Thompson* isolates is the key node of these isolates, it composed the backbone of cgMLST diagram with *S. enteritidis*, *S. typhimurium* ST34 and *S. London*. Among them, *S. enteritidis* and *S. typhimurium* presented a more important role, because all the other isolates with different serovars or STs were branched from them, 13 from *S. enteritidis*, and 6 from *S. typhimurium* (Figure 2).

### 3.3 Genotypic and phenotypic of antimicrobial resistance

**Figure 3:**
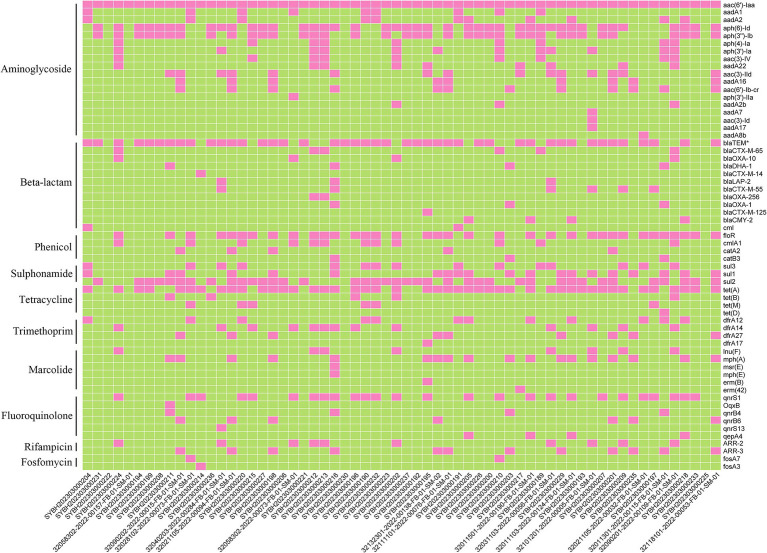
The heatmap of antimicrobial resistance genes (ARGs) in the studied *Salmonella* isolates.

Now it is easy to screen the encoded antimicrobial resistance genes (AMRGs) using whole genome sequencing. A total number of 61 different AMRGs belong to 10 different antimicrobial categories were detected. Except the *aac (6′)-Iaa* gene presented in all *Salmonella* isolates, the other most prevalent AMRG is *tet(A)* (45/62, 72.58%), *blaTEM* (44/62, 70.97%), *aph(6)-Id* (42/62, 67.74%) and *aph(3″)-Ib* (37/62, 59.68%) (Figure 3), and for the antimicrobial categories is Aminoglycoside (62/62, 100%), Beta-Lactam (56/62, 90.32%), Sulphonamide (53/62, 85.48%) and Tetracycline (49/62, 79.03%) (Table 3). The antimicrobial phenotype was also tested, and most of the phenotype could correspond to the genotype (Table 4).

**Table 3:** Prevalence of antimicrobial resistance genes in 62 *Salmonella* isolates.

Categories	Number of isolates	Prevalence rates
Aminoglycoside	62	100.00%
Beta-Lactam	56	90.32%
Phenicol	40	64.52%
Sulphonamide	53	85.48%
Tetracycline	49	79.03%
Trimethoprim	35	56.45%
Macrolide, Lincosamide and Streptogramin B	26	41.94%
Fluoroquinolone	37	59.68%
Rifampicin	22	35.48%
Fosfomycin	3	4.84%

**Table 4:** Antimicrobial resistance phenotype of 62 *Salmonella* isolates isolated in this study.

Isolates	AMP	AMS	IPM	TET	GEN	CHL	CT	AZM	CTX	CAZ	CFZ	CFX	CIP	NAL
32,011,103–2022-00124-FB-01-SM-01	R	R	S	R	S	R	S	R	R	R	R	R	R	S
32,011,301–2022-00115-FB-01-SM-01	R	R	S	R	R	R	S	S	I	I	R	R	R	S
32,011,501–2022-00190-FB-01-SM-01	R	R	S	R	S	R	S	R	R	R	R	R	R	S
32,021,105–2022-00032-FB-01-SM-01	R	R	S	R	S	R	R	R	S	S	R	S	I	S
32,021,105–2022-00094-FB-01-SM-01	R	R	S	R	R	R	S	R	I	I	I	S	R	R
32,028,102–2022-00073-FB-01-SM-01	R	R	S	R	R	R	S	R	S	S	R	S	R	S
32,031,103–2022-00056-FB-01-SM-01	R	R	S	R	R	R	S	S	R	R	R	S	R	S
32,040,203–2022-00284-FB-01-SM-01	R	R	S	R	R	R	S	S	R	R	R	R	R	R
32,058,302–2022-00073-FB-01-SM-01	R	R	S	R	R	R	S	R	R	R	R	I	R	R
32,058,302–2022-00157-FB-01-SM-01	R	R	S	S	S	S	S	S	S	S	S	S	S	S
32,090,201–2022-00109-FB-01-SM-01	R	R	S	R	R	R	S	R	R	S	I	S	R	R
32,090,202–2022-00015-FB-01-SM-01	R	R	S	R	R	R	S	R	S	S	I	S	R	R
32,101,201–2022-00005-FB-01-SM-01	R	I	S	R	R	R	S	R	R	R	R	S	R	R
32,111,101–2022-00076-FB-01-SM-01	R	R	S	R	R	R	S	R	S	S	R	S	S	S
32,118,101–2022-00053-FB-01-SM-01	R	R	S	R	R	R	S	R	S	S	R	S	R	S
32,132,301–2022-00138-FB-01-SM-02	R	R	S	R	S	R	S	R	S	S	S	S	R	S
32,010,401–2022-00012-FB-01-SM-01	R	S	S	S	I	R	S	S	R	S	I	S	I	R
32,010,501–2022-00071-FB-01-SM-01	R	I	S	R	S	R	S	S	S	S	I	S	I	S
32,011,401–2022-00069-FB-01-SM-01	R	I	S	R	S	S	S	S	S	S	I	S	S	S
32,011,401–2022-00068-FB-01-SM-01	R	R	S	S	S	S	S	S	S	S	I	S	I	R
32,011,602–2022-00096-FB-01-SM-01	R	R	S	R	S	S	S	S	S	S	I	S	I	R
32,011,501–2022-00209-FB-01-SM-01	R	R	S	R	S	S	S	S	S	S	R	S	I	R
32,011,401–2022-00099-FB-01-SM-01	R	R	S	R	R	R	S	R	R	S	I	S	I	S
32,010,401–2022-00058-FB-01-SM-01	R	I	S	R	S	R	S	S	S	S	R	S	I	S
32,010,401–2022-00150-FB-01-SM-01	R	R	S	R	S	R	S	S	R	R	I	S	I	S
32,010,602–2022-00180-FB-01-SM-01	R	R	S	R	R	R	S	S	S	S	I	S	R	S
32,010,602–2022-00134-FB-01-SM-01	R	R	S	R	S	S	S	S	S	S	S	S	I	R
32,010,501–2022-00066-FB-01-SM-01	R	R	S	R	S	S	S	S	S	S	I	S	I	R
32,010,501–2022-00059-FB-01-SM-01	R	I	S	R	R	S	S	R	S	S	R	S	S	S
32,011,101–2022-00713-FB-01-SM-01	R	R	S	R	R	R	S	S	S	S	R	S	I	S
32,031,103–2022-00086-FB-01-SM-01	R	R	S	R	S	S	S	R	R	R	I	R	R	S
32,031,103–2022-00089-FB-01-SM-01	R	I	S	R	S	S	S	S	S	S	R	S	S	S
32,031,103–2022-00104-FB-01-SM-01	R	R	S	R	S	R	S	R	R	R	R	R	R	S
32,031,103–2022-00116-FB-01-SM-01	R	R	S	R	S	S	R	S	S	S	R	S	I	R
32,031,103–2022-00125-FB-01-SM-01	R	I	S	R	S	R	S	S	S	S	R	S	I	S
32,031,103–2022-00118-FB-01-SM-01	R	R	R	S	S	S	R	S	S	R	R	R	I	R
32,031,103–2022-00119-FB-01-SM-01	R	I	S	R	R	R	S	S	R	R	R	S	I	S
32,031,103–2022-00129-FB-01-SM-01	R	S	S	R	I	R	S	S	R	S	R	S	I	S
32,031,103–2022-00136-FB-01-SM-01	R	R	S	R	S	R	S	S	R	R	R	R	I	S
32,031,103–2022-00139-FB-01-SM-01	R	R	S	R	R	R	S	S	R	S	R	S	I	S
32,031,103–2022-00140-FB-01-SM-01	R	R	S	R	R	R	S	S	R	S	R	S	I	S
32,031,103–2022-00141-FB-01-SM-01	R	R	S	R	S	S	R	S	R	S	R	S	R	R
32,031,103–2022-00143-FB-01-SM-01	R	R	S	R	R	R	R	S	S	S	R	S	R	R
32,031,103–2022-00144-FB-01-SM-01	R	R	R	R	R	R	S	R	R	R	R	R	R	S
32,031,103–2022-00148-FB-01-SM-01	R	R	S	S	R	S	R	R	S	S	R	S	I	R
32,031,103–2022-00149-FB-01-SM-01	R	R	R	R	S	R	S	R	R	R	R	R	R	S
32,028,102–2022-00059-FB-01-SM-01	R	R	S	R	S	R	S	S	S	S	R	S	I	S
32,021,105–2022-00090-FB-01-SM-01	R	R	S	S	S	S	R	S	S	S	I	S	I	R
32,028,201–2022-00080-FB-01-SM-01	R	I	S	R	R	R	S	R	S	S	I	S	S	S
32,028,201–2022-00086-FB-01-SM-01	R	R	S	S	S	S	S	S	S	S	R	S	I	R
32,028,102–2022-00072-FB-01-SM-01	R	R	S	R	R	R	S	S	R	S	I	S	I	S
32,028,102–2022-00076-FB-01-SM-01	R	S	S	R	R	R	S	S	S	S	I	S	I	S
32,028,102–2022-00083-FB-01-SM-01	R	R	S	R	S	S	S	S	S	S	I	S	I	R
32,028,102–2022-00086-FB-01-SM-01	R	R	S	R	S	S	S	S	S	S	R	S	I	R
32,021,105–2022-00035-FB-01-SM-01	R	I	S	R	R	S	S	R	S	S	R	S	S	S
32,021,105–2022-00118-FB-01-SM-01	R	I	S	R	S	R	R	S	S	S	I	S	I	R
32,021,105–2022-00003-FB-01-SM-01	R	R	S	S	S	S	S	S	S	S	I	S	I	R
32,021,105–2022-00075-FB-01-SM-01	R	I	S	R	S	R	S	S	S	S	I	S	I	S
32,021,105–2022-00080-FB-01-SM-01	S	S	S	R	I	R	S	S	S	S	I	S	I	S
32,021,105–2022-00113-FB-01-SM-01	S	R	S	S	S	S	S	R	S	S	R	S	R	R
32,028,102–2022-00101-FB-01-SM-01	R	I	S	R	S	S	S	S	S	S	R	S	S	S
32,021,105–2022-00104-FB-01-SM-01	R	R	S	S	S	S	R	S	S	S	R	S	I	R

### 3.4 Relationship between antimicrobial resistance and serovar

**Figure 4:**
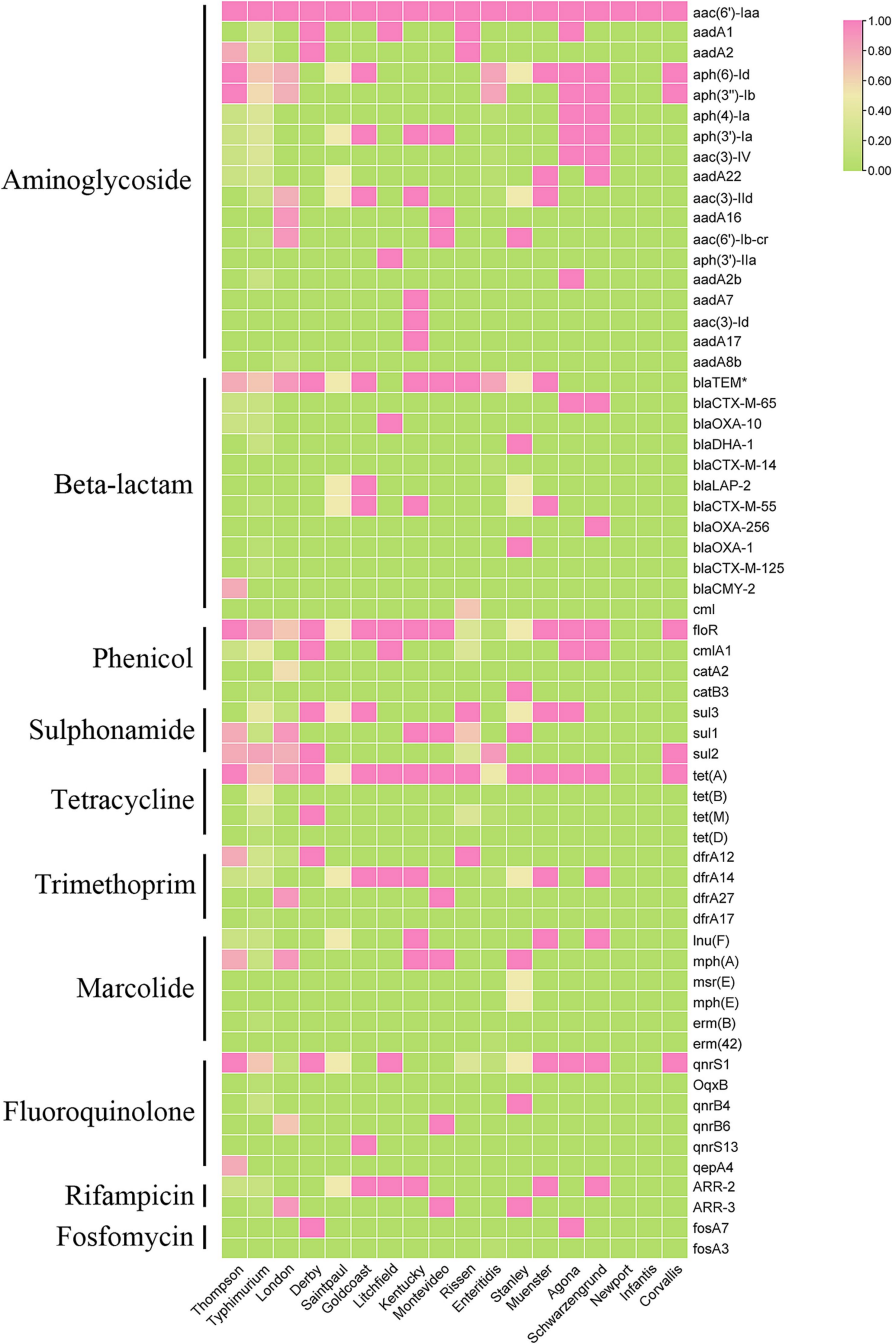
The heatmap of represent the relationship between antimicrobial resistance genes (ARGs) and serotypes of 62 *Salmonella* isolates.

In order to understand the prevalence of AMRGs among different serovars, after screened the AMRG in all those *Salmonella* isolates. Among those serovars, *S. typhimurium* harbors most diversified AMRGs (40/61, 65.57%), followed by *S. Thompson* (24/61, 39.34%) and *S. Stanley* (21/61, 34.42%). Meanwhile, there are two serovars contain only one AMRG, *aac(6′)-Iaa*, which is located on chromosome (Figure 4). This data elucidated that the prevalence of AMRGs is associated with serovars and the distribution biased.

### 3.5 Co-existence of antimicrobial resistance genes

We further analyzed that which AMRGs are prefer to exist together, considering the presence frequency of AMRGs against Aminoglycoside class drug is extremely high and the resistance gene *aac(6′)-Iaa* is located in chromosome and presence in every genome, we calculated the combinations twice, excluded *aac(6′)-Iaa* and excluded all AMRGs against Aminoglycoside class drug. *Aph(6)-Id*+*aph(3″)-Ib*+*sul2* (31 times) is most frequent when only exclude *aac(6′)-Iaa*, followed by *aph(6)-Id*+*aph(3″)-Ib*+*blaTEM* (28 times) and *aph(6)-Id*+*blaTEM*+*sul2* (27 times), we can see that these combinations are all composed by Aminoglycoside AMRGs with a Beta-Lactam AMRG *blaTEM* and/or a Sulphonamide AMRG *sul2*. When exclude the AMRGs against Aminoglycoside class drug, the most frequent combination is *blaTEM*+*floR*+*tet(A)* (24 times), equal with *floR*+*tet(A)* + *qnrS1* (24 times) and *blaTEM*+*sul2* + *tet(A)* (24 times), Tetracycline resistance gene *tet(A)* exist in every combination, and we can also found Phenicol resistance gene *floR* and Fluoroquinolone resistance gene *qnrS1* (Table 5).

**Table 5:** Top 10 most frequent antimicrobial resistance gene combinations of 62 *Salmonella* isolates (The elements in the combination > = 3).

Combination types	Frequency
Exclude *aac(6’) - Iaa*	
*aph(6)-Id + aph(3″)-Ib + sul2*	31
*aph(6)-Id + aph(3″)-Ib + blaTEM*	28
*aph(6)-Id + blaTEM + sul2*	27
*aph(3″)-Ib + blaTEM + sul2*	27
*aph(6)-Id + aph(3″)-Ib + blaTEM+sul2*	27
*aph(6)-Id + aph(3″)-Ib + tet(A)*	26
*blaTEM +f loR + tet(A)*	24
*floR + tet(A) + qnrS1*	24
*blaTEM + sul2 + tet(A)*	24
*aph(6)-Id + blaTEM + tet(A)*	23
*Exclude Aminoglycosides*	
*blaTEM + floR + tet(A)*	24
*floR + tet(A) + qnrS1*	24
*blaTEM + sul2 + tet(A)*	24
*floR + sul2 + tet(A)*	19
*blaTEM + floR + sul2*	17
*blaTEM + floR + qnrS1*	16
*blaTEM + tet(A) + qnrS1*	16
*blaTEM + floR + tet(A) + qnrS1*	16
*blaTEM + floR + sul2 + tet(A)*	16
*blaTEM + sul1 + tet(A)*	15

### 3.6 Plasmid replicons

**Figure 5:**
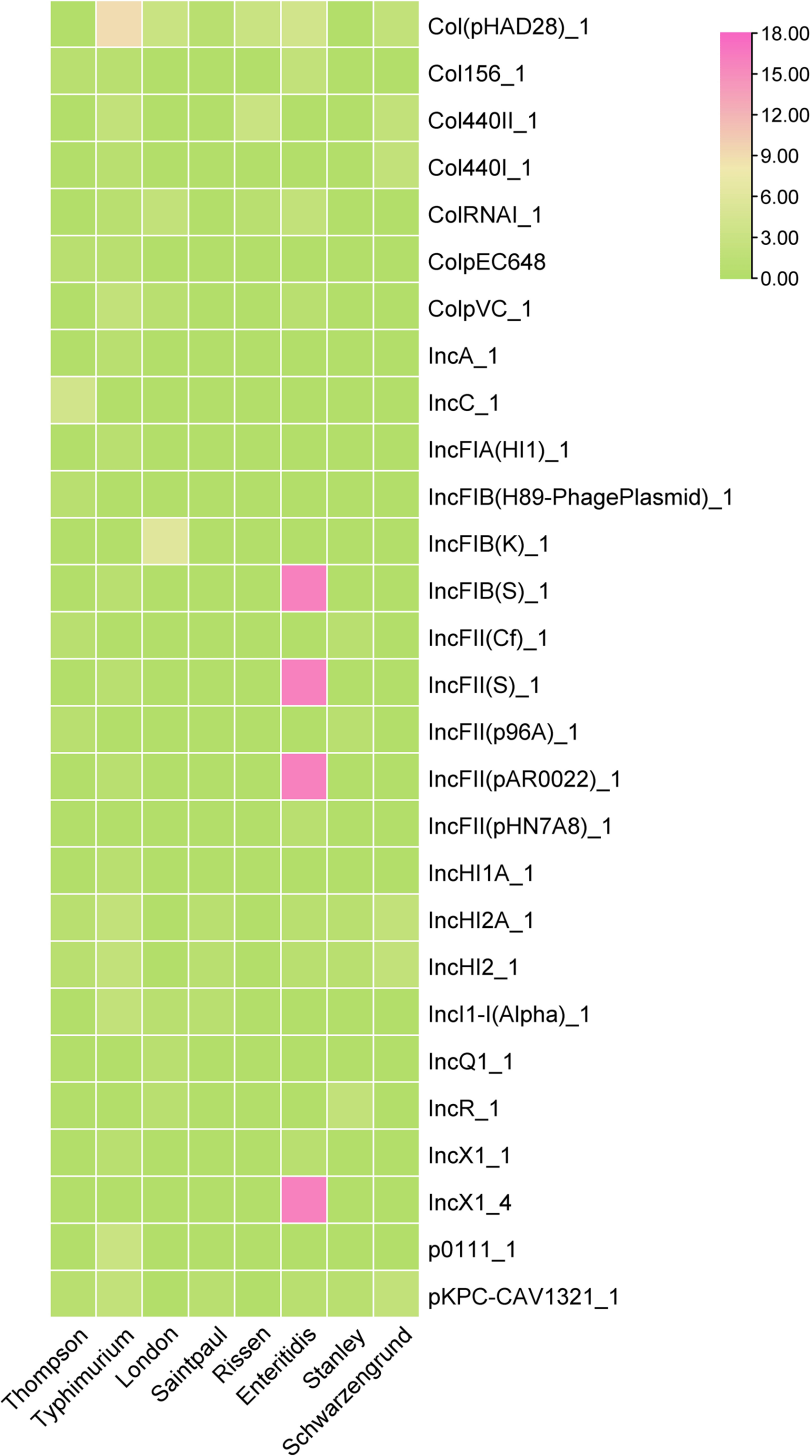
The heatmap of plasmids distribution in different *Salmonella* isolates. The strength of the colors corresponds to the numerical value of the prevalence of the plasmids.

Plasmid carrying AMRGs is one of the reasons that the spread of antimicrobial resistance, among 62 *Salmonella* isolates, 58 of them were detected carrying at least one plasmid (58/62, 93.55%), 5 of them even carrying 6 different plasmids. 28 different plasmids were detected, where the plasmid Col(pHAD28)_1 was the most prevalent (22/62, 35.48%), followed by IncFIB(S)_1, and IncFII(S)_1, IncFII(pAR0022)_1 (17/62, 27.42%) and IncX1_4 (16/62, 25.81%). Further, we found that *S. typhimurium* harbors more diversified plasmid (*n* = 19) compared with other serovars, followed by *S. enteritidis* (*n* = 13). However, only 8 of 18 serovars were identified harboring plasmids. Notably, the co-occurrence between plasmid replicons and antimicrobial resistance genes was mainly observed in *S. typhimurium* (Figure 5) 0.3.7 Characterization of plasmids carrying multiple AMRGs.

**Figure 6:**
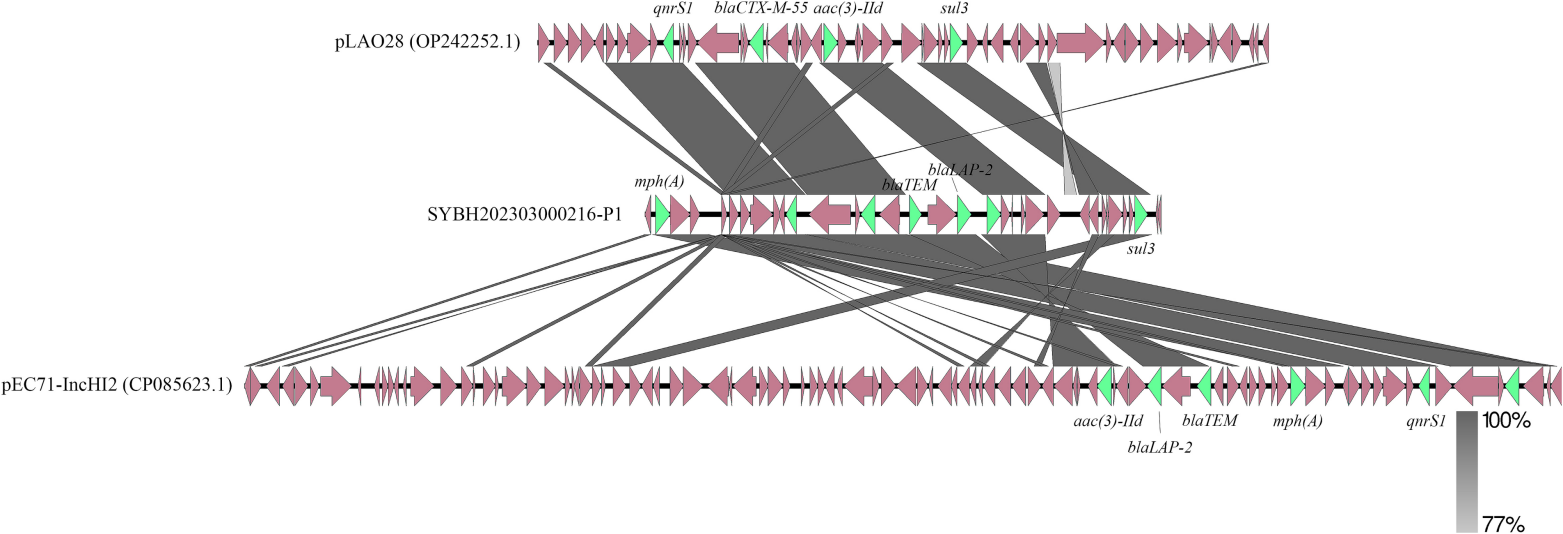
Genetic comparison of the structure of SYBH202303000216-P1 with pLAO28 (Accession No. OP242252.1) and pEC71-IncHI2 (Accession No. CP085623.1). The antimicrobial resistance genes are shown in green and the rest of the genes are shown in reddish brown.

Among these *Salmonella* isolates, a *S. Stanley* isolate contain 21 AMRGs, which is the most of them. We tried to discuss how this bacterial isolate contains that much AMRGs, through further analysis, more than half (12 of 21) AMRGs are detected located in one plasmid. The distribution of these AMRGs is wide, but still 7 of them are very close, and we compared this part of this plasmid with other plasmids with high homology, we can see that even for these closest sequences, there are still a lot of difference, rearrangement, translocation, inversion could be observed (Figure 6), The number of AMRGs in these sequences are different, the accumulation of AMRGs could be the result of gene exchange.

## 4 Discussion

A major global public health concern, non-typhoidal salmonellosis (NTS) has a huge financial and medical impact on a global scale (Paudyal et al., 2018). The present study provides a comprehensive genomic analysis of *Salmonella* isolates from outpatients in Jiangsu, China, emphasizing the genetic diversity, antimicrobial resistance (AMR) profiles, and possible processes behind the spread of resistance. Our results highlight the serious public health issues that NTS presents, especially in light of the growing worldwide burden of foodborne diseases and multidrug resistance (MDR).

The dominance of *S. enteritidis* (27.42%) and *S. typhimurium* (19.35%) among the 62 isolates aligns with global epidemiological trends, where these serovars are frequently associated with foodborne transmission and human salmonellosis (Majowicz et al., 2010). The detection of 18 serovars and 21 sequence types (STs) reflects substantial genetic diversity, consistent with the high adaptability of *Salmonella* (Cheng et al., 2019). Notably, the prevalence of ST11 (related with *S. enteritidis*) and ST34 (associated with *S. typhimurium*) was high, which is consistent with findings from other areas where these STs are connected to MDR outbreaks and phenotypes (Balasubramanian et al., 2019; Woh et al., 2025), further corroborates findings from the Chinese Local *Salmonella* Genome Database and other studies, which highlights ST34 as a pandemic MDR clone responsible for pediatric infections and livestock-associated outbreaks (Wang et al., 2023a,b). The serotype-specific genomic evolution impacts epidemiological trends comes from the phylogenetic grouping of isolates by serovar (Yoshida et al., 2016).

When the antimicrobial resistance genes in those NTS isolates were screened using the WGS technique, 61 genes encoding resistance to 10 categories were found. Except of the *aac(6′)-Iaa* gene, which confers intrinsic aminoglycoside resistance, is 100% harboring the chromosome of NTS isolates, high prevalence of *tet(A)* (72.58%), *blaTEM* (70.97%), and sulfonamide resistance genes (85.48%) was detected, underscores the widespread use of *β*-lactams, tetracyclines, and sulfonamides in clinical and agricultural settings (Alekshun and Levy, 2007; Ramirez and Tolmasky, 2010; Nazir et al., 2025). One of the most important public health concerns is the increase of *Salmonella* isolates that are resistant to antibiotics (Eng et al., 2015). Alarmingly, 90.32% of isolates had β-lactam resistance genes, which is indicative of the increase in *Salmonella* that produce extended-spectrum β-lactamase (ESBL) worldwide, reflect a national crisis in antibiotic misuse, as reported in studies analyzing >35,000 *Salmonella* isolates across China (Wang et al., 2023a). Therapeutic choices are severely limited by such developments, especially in areas where antibiotic abuse is prevalent (Okeke et al., 2024).

The serovar-dependent AMR patterns shown here underscore the relevance of clonal proliferation in resistance diffusion. *S. typhimurium* isolates had the most varied AMRGs (40/61), consistent with its status as an MDR “high-risk” clone (Pitti et al., 2023; Medalla et al., 2016). Conversely, some serotypes, like *S. infantis* and *S. Newport*, showed fewer resistance genes. This discrepancy might be due to the limited number of isolates, but *S. enteritidis*, the other dominant serotype, also only has barely half of the *S. typhimurium* resistance genes (17/61). This data implies that horizontal transmission of the AMRG containing genetic components may change depending on the serovar.

The co-occurrence of *blaTEM*, *sul2*, *aph(6)-Id*, and *aph(3″)-Ib* in more than 50% of isolates suggests that resistance genes may located closely in the genome, and these combinatorial patterns are frequently associated with mobile genetic elements (MGEs), which promote the clustering of resistance genes (Yan et al., 2024). The combination of *floR*+*tet(A)* + *qnrS1*, which was detected in 24 isolates, suggests co-selection of tetracycline, phenicol, and fluoroquinolone resistance, possibly driven by agricultural use of these antibiotics, which emphasized the importance of One Health in disease control. Given that fluoroquinolones, such as ciprofloxacin, are first-line treatments for severe salmonellosis, this occurrence makes therapy more difficult (Randall Luke et al., 2006; Chen Y. et al., 2024). The risk levels associated with antimicrobial resistance (AMR) in *Salmonella* isolates from Jiangsu are stratified by the clinical urgency of the compromised antibiotics, transmission potential of resistance genes, and the possibility of treatment failures. Resistance to *β*-lactams, particularly extended-spectrum β-lactamases (ESBLs) like *blaTEM* (detected in 70.97% of isolates), poses a critical risk (Okeke et al., 2024). Since ciprofloxacin is the first-line treatment for invasive NTS, fluoroquinolone resistance (*qnrS1* in 24 isolates) represents a high risk. Resistance compromises empirical treatment, the agricultural origin threat was highlighted by a previous study that showed a correlation between human treatment failures and qnrS1-positive *Salmonella* in poultry farms (Chen Y. et al., 2024). We found that the plasmid is widely distributed among those *Salmonella* isolates, and that the co-occurrence of MDR genes on mobile genetic elements (MGEs) increases risks by facilitating rapid horizontal gene transfer. Plasmids frequently carry integrons and transposons, which allow “resistance gene cassettes” to accumulate (Li et al., 2023).

Concern over *β*-lactam antibiotics is growing due to a combination of variables in both human and veterinary antibiotic treatments, as evidenced by the high frequency of *blaTEM* (70.97%) and ESBL-associated resistance genes. Overuse of antibiotics on farms and the release of active antimicrobials into the environment favor resistant bacterial lineages in those settings, putting humans and animals at risk of a future pandemic from an incurable bacterial infection. The misuse and overuse of *β*-lactam antibiotics in human medicine, such as prescribing them inappropriately for viral infections or not finishing treatment courses, have created sustained selective pressure that has allowed the proliferation of resistance mechanisms like β-lactamase enzymes and efflux pumps (Okeke et al., 2024).

In veterinary medicine, β-lactams are also often used to prevent illness and promote growth, frequently at subtherapeutic dosages that encourage the survival of resistant bacteria. *blaCTX-M* genes were significantly enriched in *Salmonella* by the agricultural usage of ceftiofur, a third-generation cephalosporin, in chicken farms, which then spread to humans via contaminated meat (Yang et al., 2022). Additionally, resistance is increased by lax rules on the disposal of antibiotics and environmental contamination. Residual β-lactams in agricultural and hospital wastewater generate resistance gene reservoirs, which facilitate horizontal movement between various bacterial populations (Yan et al., 2024). One Health strategy is needed to address this, which includes investing in wastewater treatment technology to stop the spread of resistance genes, prohibiting the non-therapeutic use of antibiotics in cattle, and enforcing stronger antibiotic stewardship in clinics.

We examined one isolate that had the highest amount of AMRGs among these isolates in order to better understand the horizontal transfer of antimicrobial resistance genes in *Salmonella*. The fact that over half of the AMRGs were found in a plasmid highlights the critical role that plasmids play in the spread of resistance. Plasmids containing AMRGs showed structural differences (rearrangements, inversions) that were suggestive of active recombination, according to comparative analysis. Similar results have been shown for *Salmonella* from food goods and cattle, where plasmids serve as conduits for the transmission of resistance throughout ecosystems (Li et al., 2023; Huang et al., 2025; Wang et al., 2019). The mosaic nature of these plasmids suggests ongoing evolution under antibiotic selection pressure, with implications for One Health frameworks.

The high rate of MDR *Salmonella* in Jiangsu outpatients highlights the urgent need for improved monitoring and antibiotic management. The prevalence of fluoroquinolone resistance (*qnrS1*) and ESBL-associated *blaTEM* is similar to patterns in clinical *Enterobacteriaceae*, where these genes jeopardize empirical treatment (Nüesch-Inderbinen et al., 2023; Aworh et al., 2023). *Salmonella* infection management in China, where NTS is the second most common cause of foodborne outbreaks, requires stronger laws governing the use of antibiotics in agriculture as well as better food safety procedures to break the chain of transmission from farm to fork (Chen J. et al., 2024).

As a result of clonal growth and plasmid-mediated horizontal gene transfer, our genomic study indicates a significant burden of MDR *Salmonella* in Jiangsu. The need for intersectoral interventions spanning healthcare, agriculture, and policy to slow the spread of resistant strains is highlighted by the convergence of diverse AMRGs in clinically relevant serovars such as *S. typhimurium*. To stop the spread of NTS, it is essential to improve food safety systems, invest in innovative treatments, and strengthen antimicrobial stewardship.

## Data Availability

The sequence data reported in this paper have been deposited in the Genome Sequence Archive in National Genomics Data Center, China National Center for Bioinformation/Beijing Institute of Genomics, Chinese Academy of Sciences (GSA: CRA025182) that are publicly accessible at https://ngdc.cncb.ac.cn/gsa.

## Footnotes

^1^ https://www.bioinformatics.babraham.ac.uk/projects/fastqc/

^2^ https://github.com/FelixKrueger/TrimGalore

^3^ https://github.com/tseemann/mlst

^4^ https://github.com/guogenglin/Bac_cgMLST

^5^ https://cge.cbs.dtu.dk/services/PlasmidFinder/
